# Race, Ethnicity, and Pharmacogenomic Variation in the United States and the United Kingdom

**DOI:** 10.3390/pharmaceutics15071923

**Published:** 2023-07-11

**Authors:** Shivam Sharma, Leonardo Mariño-Ramírez, I. King Jordan

**Affiliations:** 1 School of Biological Sciences, Georgia Institute of Technology, Atlanta, GA 30332, USA; shivamsharma13@gatech.edu; 2 National Institute on Minority Health and Health Disparities, National Institutes of Health, Bethesda, MD 20892, USA; marino@nih.gov

**Keywords:** pharmacogenetics, human genome, health disparities, genetic ancestry, race, ethnicity

## Abstract

The relevance of race and ethnicity to genetics and medicine has long been a matter of debate. An emerging consensus holds that race and ethnicity are social constructs and thus poor proxies for genetic diversity. The goal of this study was to evaluate the relationship between race, ethnicity, and clinically relevant pharmacogenomic variation in cosmopolitan populations. We studied racially and ethnically diverse cohorts of 65,120 participants from the United States All of Us Research Program (*All of Us*) and 31,396 participants from the United Kingdom Biobank (*UKB*). Genome-wide patterns of pharmacogenomic variation—6311 drug response-associated variants for *All of Us* and 5966 variants for *UKB*—were analyzed with machine learning classifiers to predict participants’ self-identified race and ethnicity. Pharmacogenomic variation predicts race/ethnicity with averages of 92.1% accuracy for *All of Us* and 94.3% accuracy for *UKB*. Group-specific prediction accuracies range from 99.0% for the White group in *UKB* to 92.9% for the Hispanic group in *All of Us.* Prediction accuracies are substantially lower for individuals who identified with more than one group in *All of Us* (16.7%) or as Mixed in *UKB* (70.7%). There are numerous individual pharmacogenomic variants with large allele frequency differences between race/ethnicity groups in both cohorts. Frequency differences for toxicity-associated variants predict hundreds of adverse drug reactions per 1000 treated participants for minority groups in *All of Us*. Our results indicate that race and ethnicity can be used to stratify pharmacogenomic risk in the US and UK populations and should not be discounted when making treatment decisions. We resolve the contradiction between the results reported here and the orthodoxy of race and ethnicity as non-genetic, social constructs by emphasizing the distinction between global and local patterns of human genetic diversity, and we stress the current and future limitations of race and ethnicity as proxies for pharmacogenomic variation.

## 1. Introduction

Pharmacogenomic variants are genetic differences that affect how patients respond to medications, in terms of drug efficacy and toxicity [1,2,3]. Pharmacogenomic mechanisms of action include genetic modifications to enzymes and transporters that regulate the rate at which drugs are metabolized and absorbed (pharmacokinetics) or genetic changes to drug targets (pharmacodynamics). Pharmacogenomic testing is increasingly being used to predict how individuals will respond to certain medications and to guide treatment decisions. The United States Food and Drug Administration (FDA) documents well-supported pharmacogenomic associations for 114 medications, and the Clinical Pharmacogenetics Implementation Consortium (CPIC) has developed clinical practice guidelines for 145 medications [4,5]. 

Pharmacogenomics also has implications for public health owing to differences in the frequencies of pharmacogenomic variants among population groups. A number of studies have shown differences in drug response for groups defined by race, ethnicity, and ancestry [6,7,8,9,10,11,12]. This holds for both long-used medications as well as more recently developed ones. A 2015 review found that 20% of medications approved over the preceding six years showed response differences among racial and ethnic groups [13]. Several FDA-approved drug labels now include group-specific prescription recommendations, including labels for the anticonvulsant carbamazepine, the hyperuricemic agent rasburicase, the statin rosuvastatin, and the immunosuppressant tacrolimus. Nevertheless, whether or not patients’ race or ethnicity should be routinely considered when making treatment decisions remains an open question [14,15,16,17,18].

The relevance of race and ethnicity to genetics and medicine is a topic of ongoing debate. On the one hand, race and ethnicity categories are widely considered to be social constructs with little or no biological meaning and as poor proxies for genetic diversity [19,20,21]. Furthermore, there are concerns that ascribing genetic differences to socially defined groups will reinforce outdated notions of racial difference and hierarchy [22,23,24]. On the other hand, modern genomic studies have repeatedly shown clear and observable correlations between genetic diversity and how individuals identify their race and ethnicity [25,26,27,28,29,30,31]. These correlations can be attributed to the close connections between the concepts of race, ethnicity, and ancestry, all of which define “descent-associated” groups made up of members who share characteristics based on common origins [32]. As it relates to medicine, there are numerous clinical prediction algorithms that include race-based corrections, which can yield more accurate results for minority patients but may also exacerbate health disparities [33,34,35]. 

The objective of this study was to assess the relationship between race, ethnicity, and pharmacogenomic variation. Our approach is powered by the emergence of population biobanks—prospective cohort studies that include genomic and demographic data for many thousands of participants. Genome-wide patterns of pharmacogenomic variation were captured by principal components analysis (PCA), and pharmacogenomic PCA data were used as features in machine learning classifiers to predict self-identified race and ethnicity for participants from population biobanks in the United States (US) and the United Kingdom (UK). Pharmacogenomic variation predicts participants’ race and ethnicity with high accuracy, and numerous pharmacogenomic variants show large frequency differences between race and ethnicity groups in the US and the UK. The clinical relevance of race and ethnicity to pharmacogenomic variation is underscored by the large numbers of adverse drug reactions that are predicted to occur if group-specific differences in pharmacogenomic variant allele frequencies are not accounted for.

## 2. Materials and Methods

### 2.1. Biobank Volunteer Participants

This study used data from volunteer participants enrolled in the NIH All of Us Research Program (*All of Us*) and the UK Biobank (*UKB*) [36,37]. *All of Us* participant data were accessed under the terms of the Georgia Institute of Technology Data Use and Registration Agreement, and *UKB* participant data were accessed under application number 65206. The *All of Us* operational protocol is approved by the NIH IRB (protocol number 2016-05), and ethics approval for *UKB* was obtained from the Community Health Index Advisory Group (CHIAG) for Scotland, the Patient Information Advisory Group (PIAG) for England and Wales, and the North West Multi-centre Research Ethics Committee (MREC) for the United Kingdom (project ID 299116). Written informed consent was obtained from all participants. *All of Us* participant inclusion criteria include adults aged 18 and older, the legal authority and decisional capacity to consent, and currently residing in the US or a territory of the US. *All of Us* exclusion criteria exclude minors under the age of 18 and vulnerable populations (prisoners and individuals without the capacity to give consent). *UKB* participant inclusion criteria include adults aged 40–69 at recruitment, the capacity to consent, and living within 20–25 miles of one of the *UKB* assessment centers. *UKB* exclusion criteria exclude participants who express the view that they would want to be withdrawn should they lose mental capacity or die. The main difference in inclusion criteria for the two studies relates to the age of the participants. *All of Us* includes adults aged 18 and over, whereas *UKB* includes adults aged 40–69. This difference reflects the *UKB’s* decision to focus on complex diseases of middle and old age. 

### 2.2. Biobank Participant Data

*All of Us* participant data were accessed and analyzed using the Researcher Workbench, and *UKB* participant data were downloaded from the *UKB* data portal and analyzed locally. Whole genome sequence variant data for *All of Us* participants were accessed from the Controlled Tier dataset v6 (curated version C2022Q2R2), and genotype imputed whole genome variant data for *UKB* participants were accessed from data field 21008. *All of Us* participants’ self-identified race and ethnicity data were accessed from the Controlled Tier dataset, and *UKB* participants’ self-identified ethnic group data were accessed from data field 2100. The top five largest race/ethnicity groups were taken for each biobank.

### 2.3. Pharmacogenomic Variants

Pharmacogenomic variants that are associated with patient drug response were mined from the PharmGKB database [38]. NCBI dbSNP variant identifiers (rsIDs), associated genes and drugs, and levels of evidence for variant-drug associations were taken for each pharmacogenomic variant. PharmGKB variant rsIDs were used to extract pharmacogenomic variants from *All of Us* and *UKB* whole genome variant datasets. Pharmacogenomic variant alternate allele frequencies for *All of Us* and *UKB* were calculated using PLINK v1.9 [39].

### 2.4. Machine Learning Prediction

Principal component analysis (PCA) was performed on the *All of Us* and *UKB* pharmacogenomic variants using the FastPCA program implemented in PLINK v2.0, run with the “approx” modifier for the top 25 principal components (PCs) [40,41]. Pharmacogenomic PCA data were used to predict participant race and ethnicity using machine learning classifiers, with race/ethnicity as class labels and the top 25 PC-values as feature vectors. K-nearest neighbors (k-NN), random forest (RF), and support vector machine (SVM) classifier methods were implemented using the scikit-learn machine learning library v1.1.2 for Python [42]. All three methods were implemented with randomized searches to determine optimal prediction hyperparameters (training) and 5-fold cross-validation (CV) to measure prediction accuracy (testing). Accuracy was quantified as the mean ± standard deviation for the percentage of correct race/ethnicity predictions in the five test datasets for each biobank cohort. Model training and testing were repeated for feature vectors covering contiguous ranges of 2–25 PCs. Additional details on the machine learning classification approaches used here can be found in the Supplementary Methods.

Pharmacogenomic PCA allele weights were calculated by FastPCA in the form of j=1−n variant (SNP) allele dosage coefficients for the ith PCs using ${PC}_{i}=\sum_{j=1}^{n}{AlleleWeight}_{ij}{SNPdosage}_{ij}$. The magnitude of allele weights corresponds to the effect each SNP has on a given PC, which can be taken as a measure of genetic divergence.

### 2.5. Predicted Adverse Drug Reactions

The predicted number of excess adverse drug reactions per 1000 patients for *All of Us* minority racial and ethnic group participants compared to participants from the majority White group were calculated based on toxicity-associated pharmacogenomic variant effect allele frequency differences between groups, considering the mode of effect as recessive (two toxicity effect alleles needed) or dominant (one or two toxicity effect alleles needed). For the recessive model of adverse drug reactions ($\hat{R_{ADR}}$):

$$\hat{R_{ADR}}=\left(p_{min}^{2}-p_{maj}^{2}\right)*1000$$

where $p_{min}^{2}$ is the homozygous genotype fraction for the minority group toxicity-associated allele p, and $p_{maj}^{2}$ is the homozygous genotype fraction for the majority group toxicity-associated allele p.

For the dominant model of adverse drug reactions ($\hat{D_{ADR}}$):

$$\hat{D_{ADR}}=\left(p_{min}^{2}-p_{maj}^{2}\right)*1000+\left(2*p_{min}*(1-p_{min})-2*p_{maj}*(1-p_{maj})\right)*1000$$

where $2*p_{min}*(1-p_{min})$ is the heterozygous genotype fraction for the minority group toxicity-associated allele p, and $2*p_{maj}*(1-p_{maj})$ is the heterozygous genotype fraction for the majority group toxicity-associated allele p.

## 3. Results

### 3.1. Race and Ethnicity in the All of Us and UKB Cohorts

*All of Us* and *UKB* volunteer participants self-identify their race and ethnicity upon enrollment. *All of Us* race and ethnic groups are defined based on the US Census standards, and *UKB* ethnic groups are defined based on the UK National Health Service (NHS) standards. The US makes a distinction between race based on ancestral origins, and ethnicity based on shared culture, whereas the UK defines ethnicity based on shared national origins. The race and ethnic groups are similar for both countries, albeit with differences that reflect the distinct patterns of immigration and resulting demographic characteristics of each country. For example, the Hispanic ethnic category exists only in the US, and the Asian category in the UK covers South Asian immigrants from Bangladesh, India, and Pakistan, with Chinese broken out as a separate group. The US classification allows for the selection of More than one group, whereas the UK classification requires the selection of a single ethnic group but includes a Mixed category. 

The *All of Us* participant cohort is 54.0% White, 19.6% Black or African American, 15.9% Hispanic or Latino, 3.1% Asian, and 3.6% More than one; the *UKB* participant cohort is 94.4% White, 1.9% Asian, 1.5% Black, 0.3% Chinese, and 0.6% Mixed. Although the *All of Us* cohort is substantially more racially/ethnically diverse than *UKB*, White participants make up the majority of each biobank, which could bias machine learning classification algorithms. Accordingly, White participants were randomly down-sampled to 20,000 participants for *All of Us* and 10,000 participants for *UKB* to yield more balanced group sample sizes for subsequent machine learning prediction (Table 1). Both biobanks have more female than male participants; the *All of Us* cohort is 60.8% female and *UKB* is 53.4% female. The average age for both biobanks is 53 years. 

### 3.2. Pharmacogenomic Variation, Race, and Ethnicity

**Figure 1:**
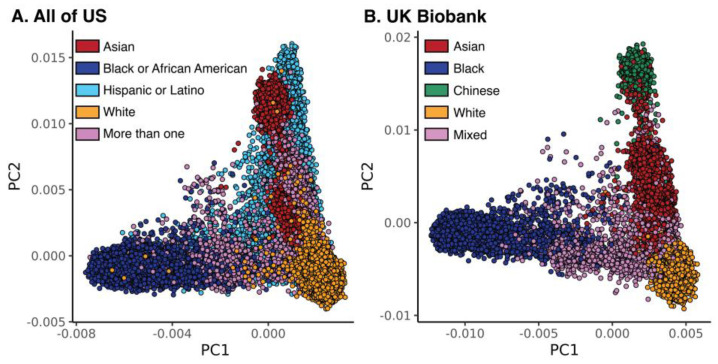
Pharmacogenomic variant principal component analyses (PCAs) for (**A**) *All of Us* and (**B**) *UKB*. Circles represent individual participants, color-coded as shown by their self-identified race or ethnicity.

Pharmacogenomic variants mined from the PharmGKB database (*n* = 6509) were intersected with genome-wide genotype data from *All of Us* (*n* = 6311) and *UKB* (*n* = 5966). Pharmacogenomic variants were analyzed using principal components analysis (PCA) and compared to self-identified race and ethnicity for *All of Us* and *UKB* participants. PCA of pharmacogenomic variants yields clusters that correspond approximately to participant race and ethnicity groups for both *All of Us* and *UKB* (Figure 1). Nevertheless, there appears to be a continuum of pharmacogenomic variation for the first two PCs with no sharp boundaries between race and ethnicity clusters. The White group forms the most coherent cluster for *All of Us*, while the White and Chinese groups are the most coherent for *UKB*. The Hispanic group has the broadest PCA distribution for any single *All of Us* group, consistent with its designation as an ethnic group that may include individuals from different racial groups. The Asian group in *All of Us* forms two clusters, corresponding to South and East Asian ancestry. The Asian group in *UKB* corresponds to South Asian ancestry, consistent with the NHS definition of the ethnic group. The More than one and Mixed groups are the most dispersed groups in *All of Us* and *UKB*, respectively.

**Figure 2:**
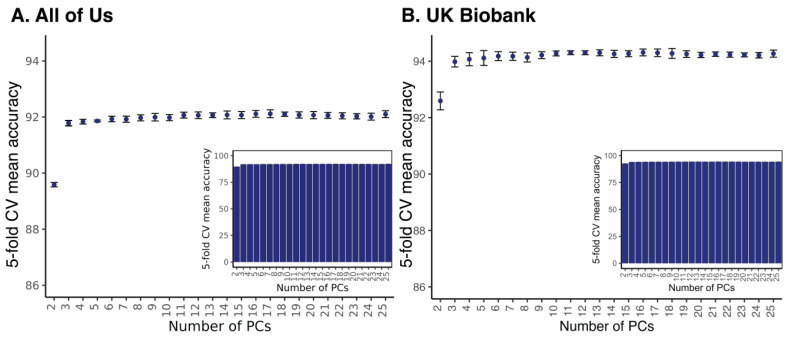
Accuracy for prediction of biobank participants’ race and ethnicity using pharmacogenomic PCA data. Results are shown for (**A**) *All of Us* and (**B**) *UKB*. Prediction accuracy values ± standard deviations, based on 5-fold cross-fold (CV) validation, are shown (*y*-axis) according to the number of PCs used for prediction (*x*-axis). The *y*-axis is truncated for clarity in the main plot, and the inset shows the same accuracy and standard deviation values with an untruncated *y*-axis.

The relationship between biobank participants’ race/ethnicity and genome-wide patterns of pharmacogenomic variation was quantified via machine learning classification. Classification algorithms are supervised learning algorithms that are used to predict categorical variables (classes) from a defined vocabulary (class labels). For this study, *All of Us* and *UKB* participants’ self-identified race/ethnicity groups were taken as class labels and pharmacogenomic PC values were taken as features used for model training and class prediction. Three different machine learning classifiers—k-nearest neighbors (k-NN), random forests (RF), and support vector machines (SVM)—were used to evaluate the accuracy with which pharmacogenomic PC values predict participant ethnicity in *UKB*. All three methods gave similar results, with the best overall performance of 94.3% mean accuracy using 16 principal components (PCs) shown by RF (Table 2). The *UKB* results for k-NN and SVM are shown in Supplementary Figure S1. Most of the pharmacogenomic variation is captured by the first 3–4 PCs (Supplementary Figure S2), and SIRE classification accuracy with RF does not change significantly after the first 3 PCs (Figure 2 and Supplementary Figure S1). The highest overall RF race/ethnicity prediction accuracy for *All of Us* is 92.1% using 17 PCs (Figure 2). 

**Figure 3:**
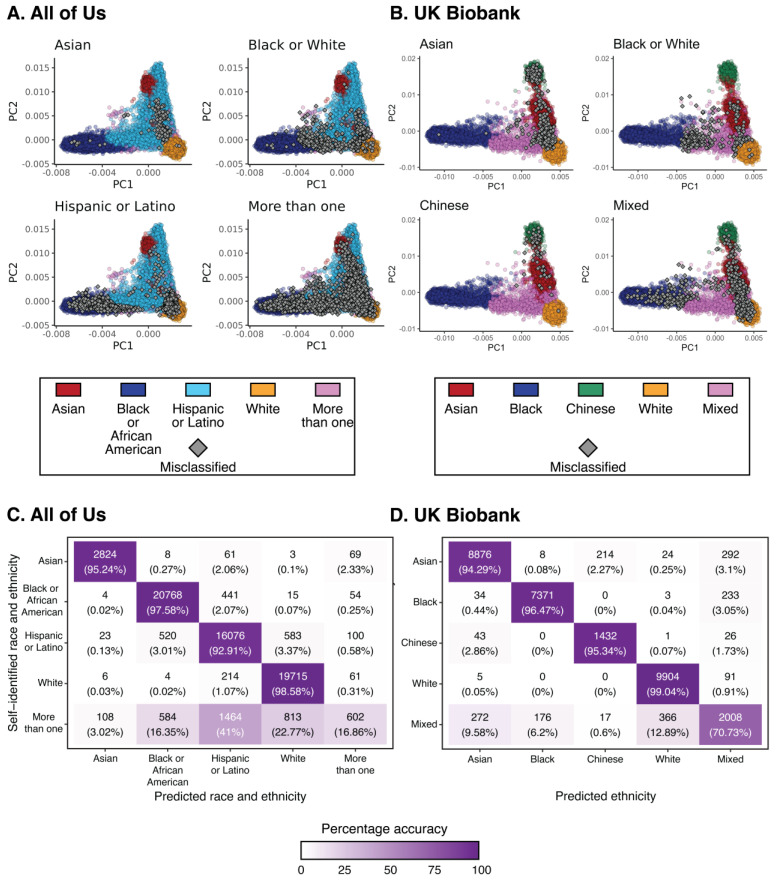
Correspondence of biobank participants’ race, ethnicity, and pharmacogenomic PCA data stratified by groups. The locations of misclassified individuals (gray diamonds) in PCA space are shown for (**A**) *All of Us* and (**B**) *UKB*. Race and ethnicity prediction accuracy values are shown for (**C**) *All of Us* and (**D**) *UKB* for all race/ethnic group combinations. Each cell shows the number and percentage of predictions for self-identified (*y*-axis) versus predicted (*x*-axis) group combinations; cells along the diagonal correspond to accurate predictions.

The accuracy of race/ethnicity classification varies according to groups in both *All of Us* and *UKB*. PC values for misclassified individuals from distinct race/ethnicity groups are shown in Figure 3A,B. Misclassified individuals from specific groups tend to map just outside the borders of their respective pharmacogenomic clusters. There is a relatively large number of misclassified Hispanic participants from *All of Us*, who tend to group with Black or White clusters, consistent with the definition of this group. Misclassified participants who identified as More than one in *All of Us* or Mixed in *UKB* show a more dispersed distribution in pharmacogenomic PC space. The accuracy of race/ethnicity prediction is highest for the White group in both *All of Us* (98.6%; Figure 3C) and *UKB* (99.0%; Figure 3D). The prediction accuracy of Hispanic individuals in *All of Us* is high (92.9%) despite the relatively high pharmacogenomic diversity of the group. Participants who identified with More than one group in *All of Us* are predicted primarily as Hispanic (41%), with broad distribution across White (22.7%), More than one (16.9%), and Black (16.3%) groups, and Mixed ethnicity is predicted with 70.7% accuracy in *UKB*. 

**Figure 4:**
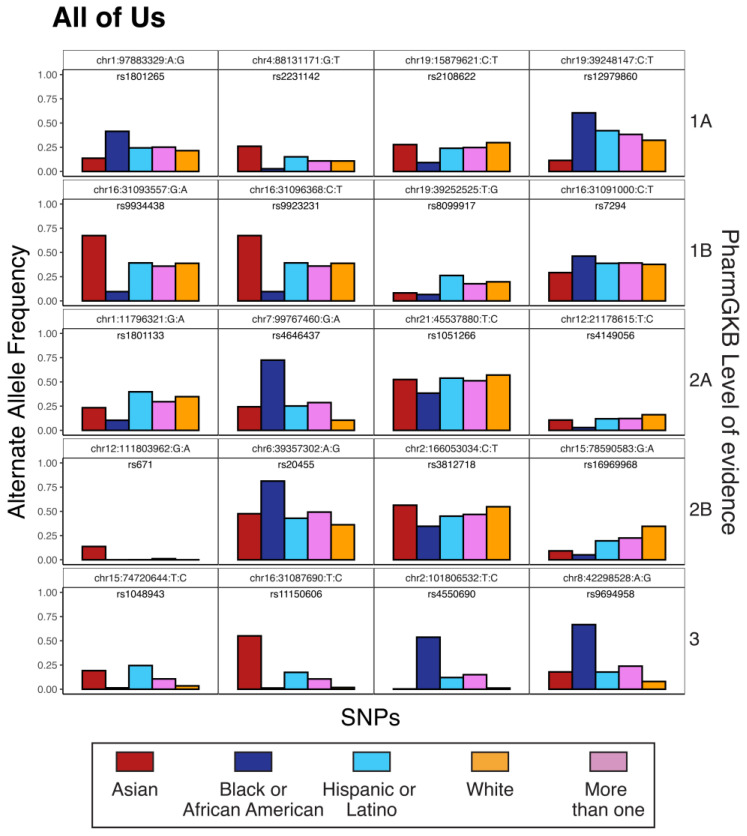
Examples of divergent pharmacogenomic variants in *All of Us*. Pharmacogenomic variants are indicated by their chromosome number, genomic position, reference allele, and alternate allele (based on GRCh38 reference genome coordinates). Variant alternate allele frequencies are shown for each race or ethnicity group. Examples are shown for the top five PharmGKB levels of evidence.

Allele weights from PC1 and PC2 were used to identify pharmacogenomic variants that have the highest levels of genetic divergence among samples (Table 3 and Supplementary Table S1). In light of the relationship between race, ethnicity, and pharmacogenomic variation, these variants tend to show the greatest allele frequency differences between race/ethnicity groups (Figure 4 and Supplementary Figure S3). Group-divergent pharmacogenomic variants of this kind can be found across PharmGKB evidence levels (1A, 1B, 2A, 2B, and 3) and correspond to effects on efficacy, dosage, and toxicity for a wide variety of drugs. 

### 3.3. Adverse Drug Reactions

The potential clinical impact of group-divergent pharmacogenomic variants was evaluated by calculating the predicted number of excess adverse drug reactions per 1000 patients for minority patients compared to the majority White group in *All of Us*. For example, the pharmacogenomic variant rs4646437 (chr7:99767460:G:A) has been associated with severe side effects among heroin-dependent patients treated with methadone [43]. The toxic effect is dominant, with both AA and AG genotype patients showing more severe side effects compared to patients with GG genotype. The A allele is found at 72.5% frequency among Black *All of Us* participants compared to 10.5% frequency for White participants. This allele frequency difference, under the dominant effect model ($\hat{D_{ADR}}$), predicts 726 more adverse drug reactions to methadone among 1000 Black patients treated compared to White patients. 

The pharmacogenomic variant rs9923231 (chr16:31096368:C:T) has been associated with the risk of anticoagulation and excess bleeding in patients treated with warfarin and phenprocoumon. The toxic effect is dominant, with CT and TT patients showing an increased risk of adverse effects. The T allele is found at 67.4% frequency among Asian *All of Us* participants compared to 33.8% frequency for White participants. This allele frequency difference, under the dominant model ($\hat{D_{ADR}}$), predicts 332 more adverse reactions to warfarin or phenprocoumon among 1000 Asian patients treated compared to White patients. 

The pharmacogenomic variant rs1801133 (chr1:11796321:G:A) has been associated with the risk of hematotoxicity among pediatric leukemia patients treated with methotrexate [44]. The adverse effect is dominant, with AA and AG genotype patients showing an increased risk of toxicity. The A allele is found at 10.4% among Black *All of Us* participants compared to 34.8% among White participants. This allele frequency difference, under the dominant effect model ($\hat{D_{ADR}}$), predicts 377 more adverse reactions to methotrexate among 1000 White patients treated compared to Black patients. 

The pharmacogenomic variant rs9694958 (chr8:42298528:A:G) has been associated with the risk of skin rash among non-small cell lung cancer patients treated with gefitinib [45]. The toxic effect is recessive, with AA genotype patients showing an increased risk of developing a skin rash. The A allele is found at 33.3% frequency among Black *All of Us* participants compared to 92.0% frequency for White participants. This allele frequency difference, under the recessive effect model ($\hat{R_{ADR}}$), predicts 735 more adverse reactions to gefitinib among 1000 White patients treated compared to Black patients. 

## 4. Discussion

The results presented here may appear to be paradoxical in light of the widely held notion that race and ethnicity are social constructs and thus poor proxies for genetic diversity. If this really is the case, then how can it be that pharmacogenomic variants predict race/ethnicity with such high accuracy, show large allele frequency differences between groups, and support the clinical relevance of race and ethnicity for adverse drug reactions? The resolution to this apparent paradox lies in the distinction between global and local patterns of human genetic diversity. The racial and ethnic group categories used in the US and the UK map poorly on global patterns of human genetic diversity, the vast majority of which are found within Africa [46,47,48]. For instance, given the extensive genetic variation and deep divergence times among African populations, White and Nigerian British individuals from *UKB* would be more closely related to each other than either is to Khoisan individuals from Southern Africa, even though Nigerian and Khoisan individuals would be racially classified as Black. There is also no reason to think that the discrete and categorical race/ethnicity groups used in the US and UK would accommodate more continuous patterns of global genetic variation [49,50]. 

Race and ethnicity, however, are defined locally in a way that reflects particular countries’ migration histories and their resulting demographic characteristics. This can be seen in the categories used by the US and UK biobanks studied here, which differ in ways that capture distinct aspects of each country’s demography. Racial and ethnic categories also change over time in a way that reflects changing demographic patterns within countries. The US census racial and ethnic classifications have changed 20 times since they were first used in the 18th century and are likely to change with the next census to reflect the increasing diversity of the country [51]. The local (and temporal) correspondence between race, ethnicity, and demography explains the connection between race, ethnicity, and genetic diversity reported here and elsewhere. This is especially true given the fact that race in the US is explicitly defined in terms of ancestral origins and ethnicity in the UK is defined in terms of immigrants’ national origins. The discontinuous sampling of divergent migrant and native populations that created modern, cosmopolitan populations, such as the US and the UK, is expected to yield clear genetic differences between socially defined groups. It is thus simultaneously true that race and ethnicity are poor proxies for global patterns of human genetic diversity, while there are also pronounced and clinically relevant genetic differences between locally defined racial and ethnic groups. In other words, socially constructed race and ethnicity groups can show genetic differences that are relevant to health. 

### Caveats and Limitations

The race and ethnicity categories studied here amount to broad groups, which may encompass multiple genetically diverged subgroups. For example, the Asian category in *All of Us* includes both East and South Asian groups, which are genetically diverged, whereas the Asian category in the *UKB* includes primarily South Asian groups. The inclusion of East and South Asian groups together in our analysis obscures pharmacogenomic differences between them. PharmGKB has adopted a biogeographic grouping system—based on seven globally geographically defined groups—to standardize the reporting of variability in pharmacogenomic allele frequencies [52]. This system is better designed to capture global patterns of pharmacogenomic variation, including countries outside the US and the UK, than socially defined race and ethnicity groups. Nevertheless, in the clinical setting, physicians have ready access to patient race and ethnicity, whereas biogeographic ancestry would require the analysis of patient genomic data.

There are other important caveats and limitations to the reliance on self-identified, and locally defined, race and ethnicity groups as proxies for pharmacogenomic variation. Beyond genetics, race and ethnicity groups also differ with respect to social determinants of health, lifestyle, and environment, all of which can be highly relevant to patient care. As it relates to genetic factors, race and ethnicity information will be rendered useless by pharmacogenomic testing, which provides a far more accurate and direct assessment of pharmacogenomic variation and risk. If all patients had ready access to pharmacogenomic testing, patients’ race and ethnicity would be irrelevant to treatment decisions. However, tests of this kind have yet to be widely and routinely implemented, and minority individuals are currently less likely to have access to genetic data of this kind [17]. In addition, as we have shown previously, race and ethnicity serve to stratify pharmacogenomic risk among population groups rather than accurately predict specific variants for any given individual [8]. In this sense, race and ethnicity should be considered pharmacogenomic risk factors for patient stratification rather than direct diagnostic tools for predicting the presence of specific variants in individual patients. 

Finally, as demographic diversity in countries such as the US and the UK continues to increase, particularly owing to increased immigration and intermarriage, traditional racial and ethnic groups will become increasingly irrelevant to pharmacogenomic risk stratification. This is supported by the relatively low prediction accuracy values seen for the More than one group in *All of Us* and the Mixed group in *UKB*. All of these facts underscore the need to move from viable but imprecise genetic proxies—such as race, ethnicity, and ancestry—to direct measures of genetic diversity in support of more equitable precision medicine. 

## 5. Conclusions

In their updated guidance for reporting race and ethnicity, the Journal of the American Medical Association declared that “Race and ethnicity are social constructs, without scientific or biological meaning” [21]. However, as we have shown here, socially defined race and ethnicity groups show differences in the frequency of pharmacogenomic variants that are directly relevant to health care. Our results on adverse drug reactions illustrate how ignoring the pharmacogenomic implications of race and ethnicity could exacerbate health disparities that burden US and UK minority groups. The social and genetic dimensions of race and ethnicity are not mutually exclusive, and the implications of both should be considered when treating patients. Considered together, the results of this study and the caveats discussed above suggest that, at this time, patient race and ethnicity should still be considered as one among many factors when making treatment decisions. 

## Figures and Tables

**Table 1:** Study cohort demography and sample sizes.

***All of Us***						
	**All**	**Asian**	**Black or African American**	**Hispanic or Latino**	**More than One**	**White**
N	65,120	2965	21,282	17,302	3571	20,000
Age (sd)	53.63 (16.55)	48.02 (16.89)	53.00 (14.74)	49.78 (15.79)	46.04 (16.29)	59.83 (17.00)
Female %	60.79	59.53	55.26	68.55	64.72	59.45
Male %	38.21	40.00	43.26	30.64	34.42	39.81
***UKB***						
	**All**	**Asian**	**Chinese**	**Black**	**Mixed**	**White**
N	31,396	9414	1502	7641	2839	10,000
Age (sd)	53.85 (8.41)	53.30 (8.45)	52.44 (7.67)	51.91 (8.06)	51.81 (8.13)	56.65 (8.05)
Female %	53.38	45.97	62.52	57.02	62.31	53.65
Male %	46.62	54.03	37.48	42.98	37.69	46.35

**Table 2:** Machine learning model performances for *UKB*. The best prediction results, based on the optimal hyperparameter values and the optimal number of PCs, are shown for each model.

Model	Hyperparameter ^a^	Parameter Value	PCs Included	Mean Accuracy	Std. Dev.
K-Nearest Neighbors	K	10	10	91.6%	0.3%
Support Vector Machine	Kernel	Radial Basis Function	15	94.0%	0.1%
	Regularization (C)	10,000			
	Gamma	10			
Random Forest	Number of trees	400	16	94.3%	0.1%
	Maximum depth of trees	110			
	Minimum samples for leaf node	2			
	Minimum samples to split node	3			

^a^ Hyperparameter definitions are provided in the Supplementary Methods.

**Table 3:** Highly diverged pharmacogenomic variants and their gene–drug associations in *All of Us*.

Variant ID ^a^	dbSNP ID ^b^	Allele Weight (PC1, PC2) ^c^	Level of Evidence ^d^	Gene	Drug
chr1:97883329:A:G	rs1801265	1.2656, 1.039	1A	*DPYD*	capecitabine, fluorouracil
chr4:88131171:G:T	rs2231142	0.6817, 1.92	1A	*ABCG2*	rosuvastatin
chr19:15879621:C:T	rs2108622	1.1024, 0.278	1A	*CYP4F2*	warfarin
chr19:39248147:C:T	rs12979860	1.3562, 0.431	1A	*IFNL3, IFNL4*	peginterferon alfa-2a, peginterferon alfa-2b, ribavirin, telaprevir, boceprevir
chr16:31093557:G:A	rs9934438	1.5132, 1.881	1B	*VKORC1*	warfarin
chr16:31096368:C:T	rs9923231	1.5097, 1.881	1B	*VKORC1*	warfarin
chr19:39252525:T:G	rs8099917	0.8534, 0.778	1B	*IFNL3*	interferons, peginterferon alfa-2a, peginterferon alfa-2b, ribavirin
chr16:31091000:C:T	rs7294	0.4052, 0.466	1B	*VKORC1*	warfarin
chr1:11796321:G:A	rs1801133	1.1024, 0.278	2A	*MTHFR*	methotrexate
chr7:99767460:G:A	rs4646437	3.2463, 0.22	2A	*CYP3A4*	tacrolimus
chr21:45537880:T:C	rs1051266	0.8781, 0.147	2A	*SLC19A1*	methotrexate
chr12:21178615:T:C	rs4149056	0.9113, 0.169	2A	*SLCO1B1*	hmg coa reductase inhibitors
chr12:111803962:G:A	rs671	0.0322, 2.559	2B	*ALDH2*	ethanol
chr6:39357302:A:G	rs20455	2.136, 0.483	2B	*KIF6*	pravastatin
chr2:166053034:C:T	rs3812718	0.8992, 0.115	2B	*SCN1A*	carbamazepine
chr15:78590583:G:A	rs16969968	1.5382, 1.389	2B	*CHRNA5*	nicotine
chr15:74720644:T:C	rs1048943	0.3912, 4.219	3	*CYP1A1*	capecitabine, docetaxel
chr16:31087690:T:C	rs11150606	0.2794, 5.708	3	*VKORC1*	warfarin
chr2:101806532:T:C	rs4550690	3.4072, 0.786	3	*MAP4K4*	anastrozole, exemestane
chr8:42298528:A:G	rs9694958	3.2572, 0.772	3	*IKBKB*	gefitinib

^a^ Variant IDs are shown as chromosome:postion:reference allele:alternate allele. ^b^ Variant IDs from the NCBI dbSNP database https://www.ncbi.nlm.nih.gov/snp/ accessed on 15 January 2023. ^c^ Allele weights are SNP dosage coefficients for each PC and measure the magnitude of variant (SNP) effects on PC values, i.e., the level of genetic divergence for each variant (see Methods). ^d^ Level of evidence in support of the reported variant–drug association taken from the PharmGKB database.

## Data Availability

All of Us data can be accessed by registered researchers using the Researcher Workbench https://www.researchallofus.org/data-tools/workbench/. *UKB* data can be accessed via researcher agreement using the Access Management System http://amsportal.ukbiobank.ac.uk/.

## Supplementary Materials

The following supporting information can be downloaded at: https://www.mdpi.com/article/10.3390/pharmaceutics15071923/s1, Figure S1: Accuracy for prediction of UKB participants’ ethnicity using pharmacogenomic PCA data; Figure S2: Scree plots for pharmacogenomic PCAs computed using *All of Us* and *UKB*; Table S1: Highly diverged pharmacogenomic variants in *UKB*; Figure S3: Examples of divergent pharmacogenomic variants in *UKB*.
