# Supplementary material for: Race, Ethnicity, and Pharmacogenomic Variation in the United States and the United Kingdom
Source: Pharmaceutics. 2023 Jul 11;15(7):1923. doi: 10.3390/pharmaceutics15071923 (PMC10383154; doi:10.3390/pharmaceutics15071923)
Supplement: Supplementary file 1 [file pharmaceutics-15-01923-s001.zip › pharmaceutics-2475172-supplementary.pdf]

Race, Ethnicity, and Pharmacogenomic Variation in the United States and the United Kingdom

Shivam Sharma [1,2], Leonardo Mariño-Ramírez [2] and I. King Jordan [1,*]

[1] School of Biological Sciences, Georgia Institute of Technology, Atlanta, GA 30332, USA; shivamsharma13@gatech.edu
[2] National Institute on Minority Health and Health Disparities, National Institutes of Health, Bethesda, MD 20892, USA; marino@nih.gov
* Correspondence: king.jordan@biology.gatech.edu; Tel.: +1-404-385-2224

Table of Contents

Supplementary Machine Learning Methods 2

Supplementary Figure S1. Accuracy for prediction of *UKB* participants' ethnicity using pharmacogenomic PCA data 3

Supplementary Figure S2. Scree plots for pharmacogenomic PCAs computed using *All of Us* and *UKB* 3

Supplementary Table S1. Highly diverged pharmacogenomic variants in *UKB* 4

Supplementary Figure S3. Examples of divergent pharmacogenomic variants in *UKB* 5

Supplementary Machine Learning Methods

Pharmacogenomic variant data were used to predict biobank participant race and ethnicity using machine learning classifiers: K-nearest neighbors (kNN), random forest (RF), and support vector machine (SVM) classifier methods were implemented using the scikit-learn machine learning library v1.1.2 for Python [1].

Model Training: *All of Us* and *UKB* participant pharmacogenomic variant data were analyzed with principal component analysis (PCA) to yield to the top 25 PC-values. KNN, RF, and SVM classification models were trained with participants' self-identified race/ethnicity as class labels and the corresponding top 25 PC-values as feature vectors. Optimal hyperparameter values for all three classification models were obtained via randomized searches with cross-validation. There was no significant gain in accuracy when an exhaustive grid search was performed in the optimal hyperparameter ranges obtained from the randomized search. Model-specific hyperparameter values are defined below:

<u>KNN hyperparameter definitions</u>
K – number of neighbors used for classification voting decisions.

<u>SVM hyperparameter definitions</u>
Kernel – mathematical functions used to transform non-linear classification problems to linear forms for solution.
Regularization (C) – how much error can be tolerated in classification.
Gamma – used to define the distance of points to a plausible hyperplane used to find the correct hyperplane.

<u>RF hyperparameter definitions</u>
Number of trees – number of trees used for the random forest classifier.
Maximum depth of tree – maximum number of edges from the root node to the leaf node in the longest path.
Minimum samples for leaf node – minimum number of samples required for leaf nodes.
Minimum samples to split node – minimum number of samples required to split an internal node.

Model Testing: The accuracy of pharmacogenomic variant-based race and ethnicity classification was tested using 5-fold cross-validation (CV) with the optimal set of hyperparameters for each model as described above. Accuracy was quantified as the mean ± standard deviation for the percentage of correct race/ethnicity predictions in the five test data sets. Testing was repeated for feature vectors covering contiguous ranges of 2-25 PCs.

1. Pedregosa, F.; Varoquaux, G.; Gramfort, A.; Michel, V.; Thirion, B.; Grisel, O.; Blondel, M.; Prettenhofer, P.; Weiss, R.; Dubourg, V. Scikit-learn: Machine learning in Python. *the Journal of machine Learning research* **2011**, *12*, 2825-2830.

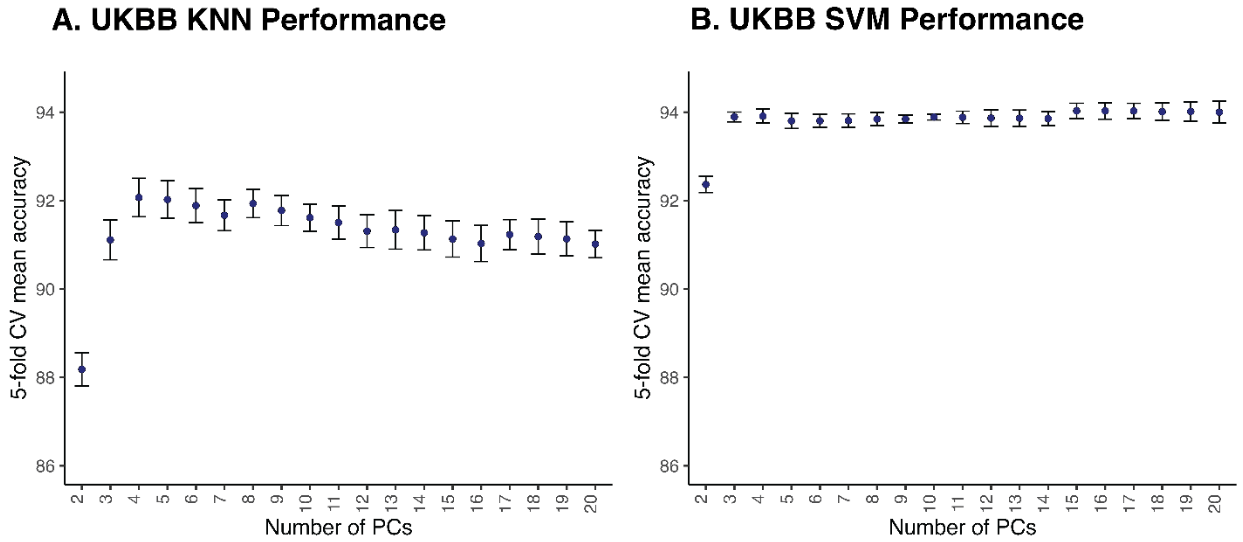

Supplementary Figure S1. **Accuracy for prediction of *UKB* participants' ethnicity using pharmacogenomic PCA data.** Results are shown for (A) k-nearest neighbors (KNN) and (B) support vector machines (SVM). Prediction accuracy values ± standard deviations, based on 5-fold cross-fold (CV) validation, are shown (y-axis) according to the number of PCs used for prediction (x-axis).

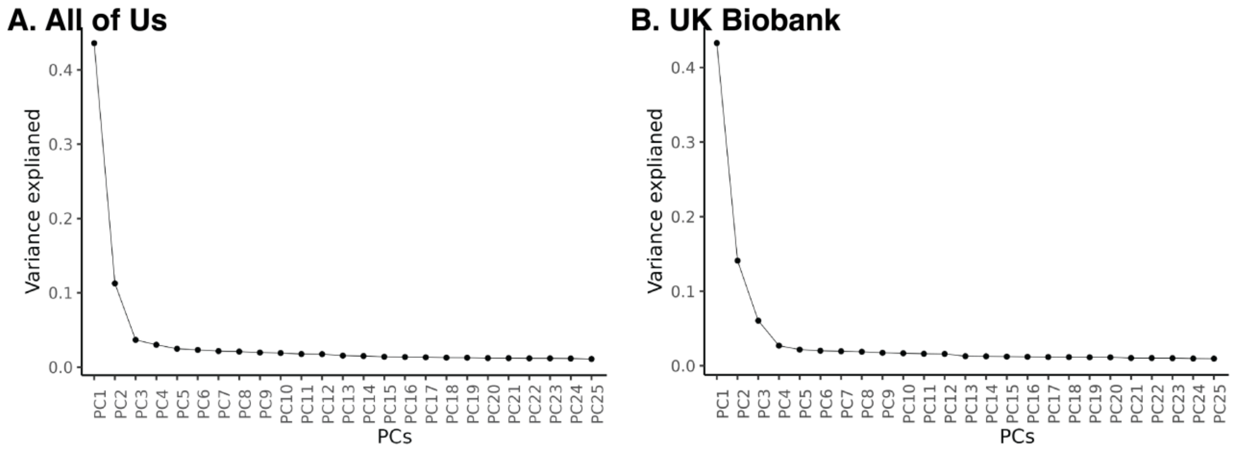

Supplementary Figure S2. **Scree plots for pharmacogenomic PCAs computed using (A) *All of Us* and (B) *UKB*.** The variance explained by each PC is shown.

Supplementary Table S1. **Highly diverged pharmacogenomic variants in *UKB*.**

dbSNP ID[a]	Allele Weight (PC1, PC2)[b]	Level of evidence[c]	Gene	Drug
rs12979860	1.686, 1.5341	1A	*IFNL3, IFNL4*	peginterferon alfa-2a, peginterferon alfa-2b, ribavirin, telaprevir, boceprevir
rs1801265	1.0063, 0.6096	1A	*DPYD*	fluorouracil, capecitabine
rs2108622	1.2262, 0.3434	1A	*CYP4F2*	warfarin
rs9923231	1.3416, 1.2879	1A	*VKORC1*	warfarin, phenprocoumon, acenocoumarol
rs7294	0.0189, 0.3776	1B	*VKORC1*	warfarin
rs2359612	0.7182, 1.1143	1B	*VKORC1*	warfarin
rs9934438	1.344, 1.2859	1B	*VKORC1*	warfarin
rs8099917	0.8142, 0.7695	1B	*IFNL3*	interferons, peginterferon alfa-2a, peginterferon alfa-2b, ribavirin
rs4646437	2.7019, 0.4643	2A	*CYP3A4*	tacrolimus
rs1051266	0.9142, 0.0781	2A	*SLC19A1*	methotrexate
rs2884737	1.1035, 1.7788	2A	*VKORC1*	warfarin
rs1801133	0.9512, 0.9922	2A	*MTHFR*	methotrexate
rs20455	2.0525, 0.2579	2B	*KIF6*	pravastatin
rs4673993	1.3894, 0.962	2B	*ATIC*	pravastatin
rs25487	1.1484, 0.1001	2B	*XRCC1*	Platinum compounds
rs3812718	1.0189, 0.412	2B	*SCN1A*	carbamazepine
rs2740574	3.8036, 0.9328	3	*CYP3A, CYP3A4*	sirolimus, cyclosporine, tacrolimus, atorvastatin, simvastatin, fentanyl, buprenorphine, lumefantrine, bleomycin, cisplatin, etoposide, atazanavir, efavirenz, cyclophosphamide, cyclosporine, carbamazepine, indinavir, docetaxel, buprenorphine, tamoxifen
rs11559290	3.3242, 1.1753	3	*ETFDH*	methylphenidate
rs6922548	3.4158, 0.8973	3	*PPARD*	docetaxel, thalidomide
rs4646450	3.1635, 0.4359	3	*CYP3A5, ZSCAN25*	tacrolimus

[a] Variant IDs from the NCBI dbSNP database https://www.ncbi.nlm.nih.gov/snp/
[b] Allele weights are SNP dosage coefficients for each PC and measure the magnitude of variant (SNP) effects on PC values, i.e. the level of genetic divergence for each variant (see Methods).
[d] Level of evidence in support of the reported variant-drug association taken from the PharmGKB database.

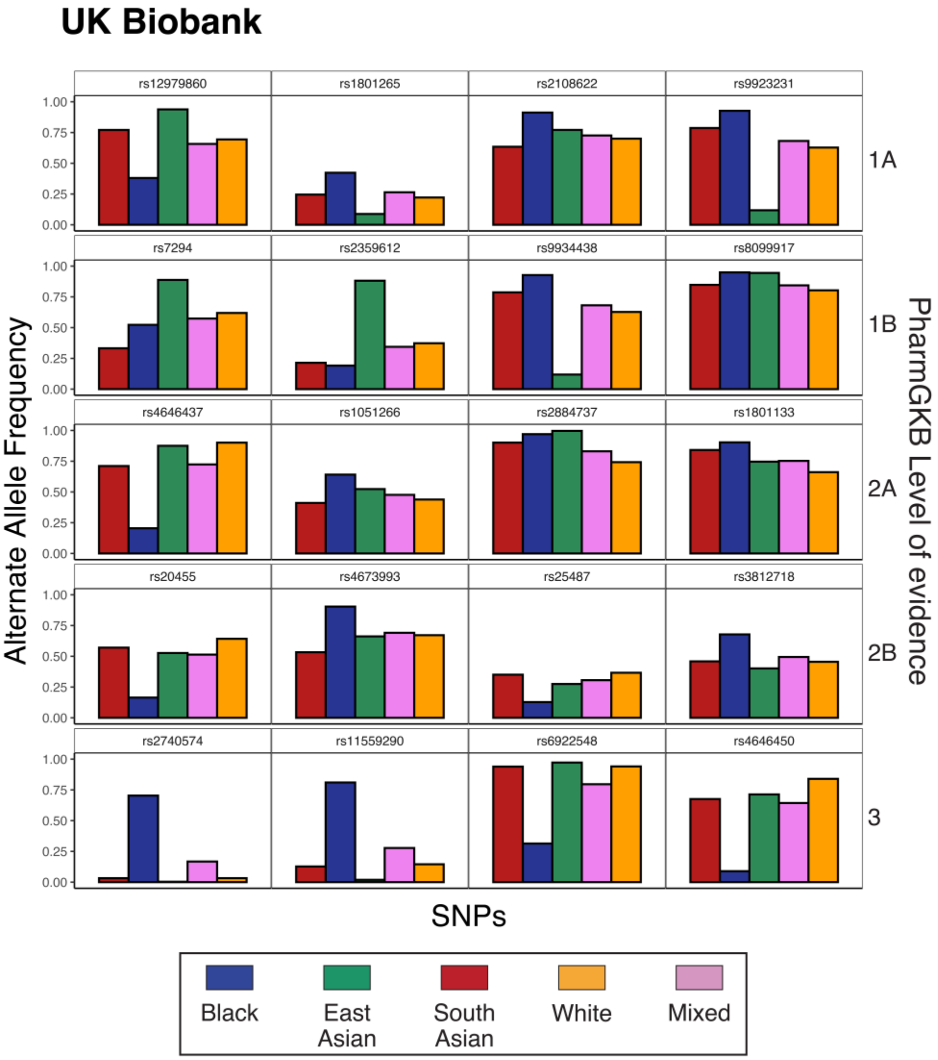

Supplementary Figure S3. **Examples of divergent pharmacogenomic variants in *UKB*.** Variant alternate allele frequencies are shown for each ethnic group. Examples are shown for the top five PharmGKB levels of evidence.
